# JBO 2023 List of Reviewers

**DOI:** 10.1117/1.JBO.29.1.010102

**Published:** 2024-01-12

## Abstract

Thanks to reviewers who served JBO in 2023.

The *Journal of Biomedical Optics* sincerely thanks the following individuals who served as reviewers in 2023. The success of our publication hinges on the voluntary contributions of time and energy put forth by these professionals.

Marwa Abdelaziz
Latifa Abdelgawad
ElhamAbouei
Tanishq Abraham
Deepshikha Acharya
Salavat Aglyamov
Ajmal Ajmal
Walter Akers
Alba Alfonso-Garcia
Julia Alonso
Stefan Andersson-Engels
Matthew Applegate
Oriol Arteaga
Çağlar Ataman
Xavier Attendu
Kamran Avanaki
Justin Baba
Wesley Baker
Tom Bakker Schut
Santosh Balakrishnan
Mihaela Balu
Timothy Baran
Luca Baratelli
Giovanni Barbera
Adam Bauer
Bernhard Baumann
Paul Beard
Athira Beena Sathyakumar
Mikhail Berezin
Renzhe Bi
Laurent Bigue
Kostadinka Bizheva
Giles Blaney
Walter Blondel
Nienke Bosschaart
Richard Bouchard
Audrey Bowden
Emil-Alexandru Brujan
Maruthi Manoj Brundavanam
David Busch
Alexander Bykov
Refik Mert Cam
Christopher Campbell
Kirby Campbell
Murat Canpolat
Jiaming Cao
Guogang Cao
Rui Cao
Xu Cao
Stefan Carp
Malte Casper
Jonathan Celli
Joseph Chaiken
Shubham Chandel
Kai-Wei Chang
Shuang Chang
Shihchi Chen
Sung-Liang Chen
Xi Chen
Xueli Chen
Yu Chen
Siyu Chen
Xiaojun Cheng
Nikita Chernomyrdin
Ata Chizari
Sang Hoon Chong
Joseph Chue-Sang
Samuel Chung
Riccardo Cicchi
Megan Clark
Lorenzo Cortese
Daniel Côté
Benjamin Cox
Michal Dabrowski
Ralph DaCosta
Michael Daly
Lisanne de Boer
Sree Bash Chandra Debnath
Savannah Decker
Hamid Dehghani
Valentin Demidov
Bin Deng
Riley Deutsch
Dinesh Dhankhar
Michele Diana
Jouke Dijkstra
Alexander Dixon
Dominik Doktor
Alexander Doronin
Alexandre Douplik
Mark Draelos
Roshan Dsouza
Andrey Dunaev
Madeleine Durkee
Nicholas Durr
Adam Eggebrecht
Jonathan Elliott
Daniel Elson
Olga Eremina
Mohsen Erfanzadeh
Thomas Ertl
Helge Ewers
Baowei Fei
Xiaohua Feng
Jinchao Feng
Farzad Fereidouni
Morgan Fogarty
Jeffrey Folz
Andrew Forbes
Ingemar Fredriksson
Daniel Fried
Liang Gao
Fei Gao
Xiaowei Ge
Elina Genina
Mina Gheiratmand
Nirmalya Ghosh
Michael Giacomelli
Holly Gibbs
Randolph Glickman
Wei Gong
Dimitris Gorpas
Debabrata Goswami
Janek Groehl
L. Jay Guo
Philipp Gutruf
Neda Haj-Hosseini
Martin Hammer
Guang Han
Katharine Hanlon
Andreas Hauptmann
Chao He
Sicong He
Wido Heeman
Herbert Heise
Eric Henderson
Kurt Hingerl
Abraham Hirshberg
Alison Hobro
Mohammad Hodaei
Jon Holmes
Xuechuan Hong
Dewei Hu
Weida Hu
Chenfei Hu
Song Hu
Kevin Huang
Gereon Huettmann
Martin Hultman
Eno Hysi
Justus Ilgner
Jared Jagdeo
Evan Jelly
Yali Jia
Mengyu Jia
Janggun Jo
Hanna Jonasson
Keiichiro Kagawa
Jana Kainerstorfer
Vyacheslav Kalchenko
Dongkyun Kang
Dimitris Kapsokalyvas
Kavon Karrobi
Deepa Kasaragod
Matthew Keller
Björn Kemper
Gordon Kennedy
Anjul Khadria
Ki Hean Kim
Matthias Klemm
Lisa Kobayashi
Ivan Kosik
Sri-Rajasekhar Kothapalli
Murali Krishnamoorthy
Nikola Krstajic
Grey Kuling
Xiaomin Lai
Puxiang Lai
Jesse Lam
Kirill Larin
Ethan LaRochelle
Vinh Nguyen Du Le
Tim Lee
Hee Ryung Lee
Youngseop Lee
Eui Chul Lee
Frédéric Lesage
Huimin Leung
Terence Leung
Richard Levenson
Changqing Li
Jiao Li
Qingli Li
Zhaohui Li
Dongyu Li
Haoyu Li
Changhui Li
Zhe Li
Shuying Li
Lei Li
Jinyang Liang
Guiqiu Liao
Adam Liebert
Liwei Liu
Yang Liu
Quan Liu
Xinwen Liu
Yang Liu
William Lo
Daniel Louie
Guolan Lu
Felix Lucka
Geoffrey Luke
Cheng Ma
Yayao Ma
Calum MacAulay
Philipp Machler
Mark Mackanos
Nicholas MacKinnon
Pierre Mahou
Boris Majaron
Oleksandr Maksymenko
Srivalleesha Mallidi
Biagio Mandracchia
Srirang Manohar
Yash Mantri
Zbignevs Marcinkevics
Haley Marks
Matthew Martell
Fabrizio Martelli
Stephen J. Matcher
Siavash Mazdeyasna
Niall McAlinden
David McClatchy
Michael McShane
Andrei Medvedev
Igor Meglinski
Guanghan Meng
Miriam Menzel
J. Sven Mieog
Eric Miller
Takeo Minamikawa
Mircea Mujat
Sabyasachi Mukhopadhyay
Andrea Murray
Oleg Nadyarnykh
Wouter Nagengast
Peter Naglic
Achuth Nair
Sreyankar Nandy
Sujatha Narayanan Unni
Meda Negrutiu
John Quan Nguyen
Thien Nguyen
Bo Ning
Izumi Nishidate
Nozomi Nishimura
Tatiana Novikova
Marien Ochoa Mendoza
Claus-Dieter Ohl
Chukwuemeka Okoro
Jeffrey Oliver
Malini Olivo
Xavier Orlik
Gregory Palmer
Francesca Palombo
Vikas Pandey
Asael Papour
Arturo Pardo
Evgueni Parilov
B. Hyle Park
Hyeon-Cheol Park
Seonyeong Park
YongKeun Park
Dan Pham
Thinh Phan
Daqing Piao
Mark Pierce
Antonio Pifferi
Anahita Pilvar
Brian Pogue
Simon Poland
Alexey Popov
Asima Pradhan
Jaya Prakash
Manojit Pramanik
Ruobing Qian
Timothy Quang
Muhammad Mohsin Qureshi
Matin Raayai Ardakani
Narasimhan Rajaram
Praveenbalaji Rajendran
Sudheesh Rajput
Julio Ramirez-San Juan
Lise Randeberg
Aniruddha Ray
Gabriel Regnault
Karen Reiser
Samuel Remedios
Liqiang Ren
Marco Renna
Demelza Robinson
Mitchell Robinson
Dominic Robinson
Jonathan Rocheleau
Eladio Rodriguez-Diaz
Stephan Rogalla
Andrew Rollins
Bakhtiar Affendi Rosdi
Bernhard Roth
H. Grady Rylander III
Rolf Saager
Teemu Sahlstrom
Partha Sahu
Guennadi Saiko
Inga Saknite
Ahmad Salman
David Sampson
Travis Sawyer
Ilyas Saytashev
Herbert Schneckenburger
Felix Scholkmann
Richard Schwarz
Augustino Scorzo
Eric Seibel
Myeongsu Seong
Ruibo Shang
Yu Shang
Yuecheng Shen
Jindou Shi
Leonid Shmuylovich
Leena Shoemaker
Ke Si
Kanwarpal Singh
Michael Singh
Manmohan Singh
Lagnojita Sinha
David Sliney
Thomas Smart
Jason Smith
Laura Sordillo
Michael Sowa
Janis Spigulis
Samuel Spink
Keith St. Lawrence
Samuel Streeter
Oliver Stumpp
Iain Styles
Jiande Sun
Tengfei Sun
Ulas Sunar
Syeda Tabassum
Bingyao Tan
Xiaodong Tao
Chao Tao
Yuankai Tao
Michaela Taylor-Williams
William Thomas
Robert Thomas
Chao Tian
Jie Tian
Karissa Tilbury
Jerilyn Timlin
Eric Tkaczyk
Veronica Torres
Jens Traeger
Minh Tran
Daniel Tso
Vassiliy Tsytsarev
Valery Tuchin
Hafeez Ullah Janjua
Paul Kumar Upputuri
Pablo Valdes
Jos Van der Hage
Ton van Leeuwen
Gijs Van Soest
Jenu Varghese Chacko
Priyanka Vasanthakumari
Georgii Vasiukov
Sandhya Vasudevan
John Viator
Umberto Villa
Martin Villiger
Sunil Vyas
Alec Walter
Peng Wang
Xiong Wang
Zhiyang Wang
Feifei Wang
Zhuoying Wang
Wenbo Wang
Adam Wax
Timothy Weber
Robert Weersink
Rene Werkmeister
Brian White
Jesse Wilson
Martin Wolf
Cayla Wood
Jiamin Wu
Min Wu
Kuancheng Wu
Yicong Wu
Jingyi Wu
Rosalind Wynne
Jun Xia
Liangzhong Xiang
Weisi Xie
Yuan Xu
Chen Xu
Guan Xu
Phaneendra Yalavarthy
Sihua Yang
Jiamiao Yang
Bin Yang
Xinmai Yang
Xuefeng Yang
Xincheng Yao
Gang Yao
Jian Ye
Qing Ye
Ji Yi
Sixian You
Linhui Yu
Shuhua Yue
Jeff Zabel
Vladimir Zaitsev
Lvming Zeng
Haishan Zeng
Ye Zhan
Limin Zhang
Wei Zhang
Yi Zhang
Yide Zhang
Jingjing Zhao
Mengyang Zhao
Yanyu Zhao
Hongting Zhao
Hubin Zhao
Jianhua Zhao
Emily Zheng
Wei Zheng
Jian Zhong
Pei Zhong
Chao Zhou
Xin Zhou
Feifan Zhou
Dan Zhu
Caigang Zhu
Jiang Zhu
Shouping Zhu
Lawrence Ziegler
Bernhard Zimmermann
Peyman Zirak
Fernando Zvietcovich

